# Muscle strength after the anterior cruciate ligament reconstruction via contralateral bone-tendon-bone autograft

**DOI:** 10.1186/s40634-021-00399-y

**Published:** 2021-10-06

**Authors:** Takaki Sanada, Eiji Uchiyama, Hiroshi Iwaso, Atsushi Fukai

**Affiliations:** 1 Department of Sports Orthopaedic Surgery, Kanto Rosai Hospital, Kanto Rosai Hospital, 1-1 Kizuki Sumiyoshicho, Nakahara-Ku, Kawasaki, Kanagawa Prefecture 211-8510 Japan; 2 Inanami Spine and Joint Hospital, Tokyo, Japan; 3 Faculty of Medical Technology, Teikyo University, Tokyo, Japan

**Keywords:** Anterior cruciate ligament, Anterior cruciate ligament reconstruction, Bone-tendon-bone, Contralateral, Contralateral graft, Contralateral reconstruction, Quadriceps muscle, Return to sports

## Abstract

**Purpose:**

The anterior cruciate ligament (ACL) reconstruction via a contralateral bone-tendon-bone (C-BTB) autograft was introduced to encourage early return to sports. The purpose of this study is to evaluate whether primary contralateral BTB ACL reconstruction can be adapted for early return-to-sports modification by investigating the chronological changes of muscle strength after surgery.

**Methods:**

Fifteen patients who had underwent C-BTB ACL reconstruction were compared with a matched group of 15 patients of ipsilateral BTB (I-BTB) ACL reconstruction. The clinical outcomes of the time of return-to-sports, Tegner activity scale and the rate of second ACL injuries, the tibial anterior translation measurement, and knee extension and flexion muscle strength were assessed.

**Results:**

Within 12 months after surgery, 14 of 15 patients from both groups returned to preinjury sports. The median time to return to sports after surgery was 6.5 months in the C-BTB group and 8.0 months in the I-BTB group (*p* = 0.021). No significant difference was noted with regard to the Tegner activity scale, reinjury rate or mean instrumental anterior tibial translation. The quadriceps muscle strength in the ACL-reconstructed knee compared with the opposite knee in both groups at 5 months after surgery was 120.6% in the C-BTB group and 70.0% in the I-BTB group (*p* < 0.001). However, the quadriceps muscle strength of the non-reconstructed limb, which instructed the graft harvested knee in the C-BTB and the intact knee in the I-BTB group, compared with that of the preoperative uninjured limb, was 74.5% in the C-BTB group and 118.7% in the I-BTB group (*p* = 0.0021) 5 months after surgery. Moreover, the quadriceps muscle strength of the reconstructed knee compared with the preoperative normal limb was 88.8% and 81.5% in the C-BTB and I-BTB groups, respectively (*p* = 0.38).

**Conclusions:**

ACL reconstruction via the C-BTB autograft indicated better quadriceps muscle strength from early stage after surgery compared with I-BTB ACL reconstruction. However, the ostensible rapid symmetrical muscle strength recovery was attributed to strength deficits compared to the preoperative condition at the donor site limb and ACL-reconstructed limb.

**Level of evidence:**

Level: Level: 4.

## Introduction

Recovery from anterior cruciate ligament (ACL) injuries in athletes, especially at pre-injury levels, is often difficult. The overall return-to-play rate ranges from 60% to 80% in various sports [7]. Furthermore, only 63% of the patients re-engage in their preinjury sports, and only 44% among competitive athletes did [1]. Consequently, it takes 6–13 months for these patients and athletes to return to sports after ACL reconstruction [9]. Generally, ACL rupture is a season-ending injury for athletes since it requires a long time for them to recover and return to playing sports.

Early return-to-play, that even increases a risk of the rate of sustaining a second ACL injury [2], is often a matter of consequence among young athletes who survive in competitive teams. To shorten the return-to-play time successfully, Shelbourne et al. developed contralateral BTB ACL reconstruction [14]. This procedure results in accelerated rehabilitation that allows patients to recover fully and return to playing sports in approximately 4–6 months after reconstruction [5, 14, 17]. The main concept of the procedure is to secure better quadriceps muscle strength by harvesting a bone-patellar tendon-bone graft from the unaffected limb, which divides the duplicative antagonistic effects of reconstruction and graft harvesting maneuvers on one limb into two separate knees. This earlier return-to-sports strategy is supported by the biological healing advantage of BTB autografts, which mature faster than hamstring grafts [6].

In the return-to-play criteria, 86% of the studies used isokinetic muscle strength tests and symmetrical quadriceps strength recovery more than 80% of that of the opposite limb set as the goal after ACL reconstruction [4, 12]. According to a previous study on the muscle strength measurement on the contralateral BTB ACL reconstruction, the patients who underwent contralateral BTB ACL reconstruction secured better quadriceps muscle strength than ipsilateral BTB ACL reconstruction at 2 years postoperatively [13]. However, no report on contralateral BTB reconstruction described muscle strength in the early period when the patients returned to play.

The purpose of the study was to evaluate whether primary contralateral BTB ACL reconstruction can be adapted for early return-to-sports modification by investigating the chronological changes of muscle strength after surgery.

## Materials and methods

This study was approved by the institutional review board of Kanto Rosai Hospital. The ID number was 2019–9. Informed consent for responding to treatment was obtained from each patient by the responding surgeon, and participant agreement in the clinical research was conducted with opt-out approval.

Between 2011 and 2014, 363 patients underwent ACL reconstruction via the autograft of the bone-patellar tendon-bone in our institution. After excluding 167 revision ACL surgeries, 18 bilateral ACL reconstructions, and 15 knees with multiple ligaments, the remaining 163 primary ACL reconstruction via the transtibial tunnel creation technique were included in the investigation. Of 163 primary BTB reconstruction knees, 15 athletes underwent ACL reconstruction surgeries using the contralateral BTB autograft (C-BTB) and 148 knees underwent ACL reconstruction with the ipsilateral BTB autograft (I-BTB). The selection for the surgical procedure via the contralateral BTB graft was determined under adequate discussions between the surgeon and the patients who wished to return to their previous sports in an earlier period. To compare the two different procedures, 15 knees from 148 primary ipsilateral BTB reconstruction patients were selected as the control matched group to decrease preoperative bias. The groups were matched for sex, age, type of sports activity, Tegner activity scale, and incident of concomitant meniscus injuries (Table 1). The mean follow-up periods were 14.9 months in the C-BTB group and 20.9 months in the I-BTB group.

**Table 1:** Matched patients’ baseline characteristics

Group	Contralateral BTB	Ipsilateral BTB	*p* value
	(n=15)	(n=15)	
Age (y) (mean [ min.-max.])	20.2 [16-36]	19.7 [14-27]	0.74
Height (cm) (mean [ min.-max.])	175.1[166-186]	174.1[163-183]	0.7
Weight (kg) (mean [ min.-max.])	75.9[53-115]	75.7[50-105]	0.98
Sex (n (%))			
Female	4 (26.7)	3 (20)	0.67
Male	11(73.3)	12 (80)	
Sports (n (%))			
American football	4 (26.7)	4 (26.7)	0.731
Soccer	1 ( 6.7)	2 ( 13.3)	
Basketball	4 (26.7)	4 (26.7)	
Rugby	4 (26.7)	4 (26.7)	
Snowboard	1 ( 6.7)	0 ( 0.0)	
Tegner activity scale (median [ min.-max.])	9 [6-10]	9 [6-10]	1
Concomitant meniscal injuries (n (%))	4 (26.7)	3(20.0)	0.67

*BTB* Bone-tendon-bone

We compared the median return-to-sports times between the two groups. The median postoperative Tegner activity scale, incidence of second ACL injuries, and the mean tibial anterior translational distance difference between both knees using the KT-2000 arthrometer (MedMetric Inc., San Diego, CA, USA), and quadriceps and hamstring muscle strengths between the two groups were investigated. In this study, we defined return to sports as participation in the normal practice of the whole team on their previous team or another team. Muscle strength was compared in three different evaluation methods using the Biodex System 3 isokinetic dynamometer (Biodex Medical Systems, New York, USA) at anglar velocities of 60°/s. First, the quadriceps and hamstring muscle strength of the reconstructed knee compared with the opposite knee were investigated. Then, the chronological muscle strength change of the reconstructed knee was measured by comparing it with the preoperative uninvolved normal knee. Third, the chronological change in muscle strength of the non-reconstructed knees, including the graft harvested donor knee in the C-BTB group and the uninvolved intact knee in the I-BTB group, was investigated. Preoperative muscle strength was measured at the day before surgery. Muscle strength was measured at the following three periods: preoperative, 5 months after surgery, and 12 months after surgery.

Reconstruction surgeries were performed using the central one-third of the bone-patellar tendon-bone autograft. The BTB grafts were harvested from the ipsilateral knee in the I-BTB group and from the contralateral knee in the C-BTB group. A single round tibial tunnel was created first; then, a round femoral tunnel socket was created via a transtibial approach. The bone-patellar tendon-bone autograft was fixed by the suspension device of the ENDOBUTTON (Smith and Nephew, Inc., Andover, MA, USA) on the femoral cortex and tied to the tibial post fixation screw and washer (MeiraGTx Limited, Nagoya, Japan). All autografts were fixed at the full knee extension position while applying adequate tension to the graft by hand.

Postoperative rehabilitation was performed using the same protocol for both groups. Physical therapy was initiated on postoperative day 1, with the patients wearing a hinged knee orthosis. Once tolerated, the patients started walking with full weight bearing. The range of motion exercise was started to aim for an immediate full extension knee from the following day and gradual flexion to obtain full flexion at 4 months after surgery. Jog training began 2 months postoperatively. Agility exercises and open kinetic muscle training were started 4 months after surgery. At 5 months postoperatively, after estimating isokinetic knee extension and flexion muscle strength, the patients participated in sports activities aiming at returning to the competitive level when they have met the goal set for quadriceps and hamstring muscle recovery. We set the goal of muscle strength to be over 80% knee extension and flexion peak torque of the reconstructed knee compared with that of the contralateral extremity using the Biodex System 3 isokinetic dynamometer.

For statistical analysis, we used EZR (Jichi Medical University, Saitama Medical Center, Saitama, Japan), which is a modified version of R (The R Foundation, Vienna, Austria) for 1:1 matching data extraction from the pooled control group and comparison datasets between the two groups. Then, we used the Mann-Whitney U test to compare the groups in ordinal and continuous variables and chi-square test for the categorical variables with Excel statistical analysis (BellCurve, version 3.21). We defined *p*-values of < 0.05 as being statistically significant in the series.

## Results

**Fig. 1:**
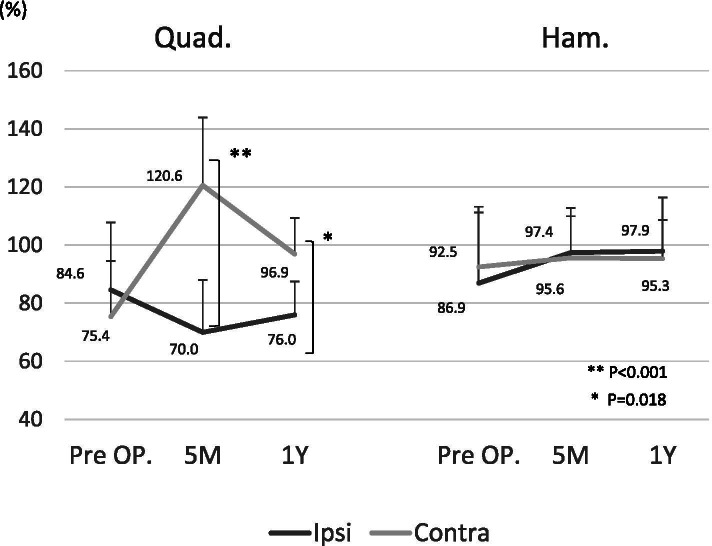
When the side-to-side muscle strength ratio were evaluated, the strength of the quadriceps and hamstring muscle was not different between the two groups preoperatively (Quad.: 75.4 ± 19% in the C-BTB vs. 84.6 ± 21% in the I-BTB; *p* = 0.194, Ham.: 92.5 ± 18.7% in the C-BTB vs. 86.9 ± 17.8% in the I-BTB; *p* = 0.49). However, quadriceps muscle strength in the C-BTB group was significantly higher than that in the I-BTB group at 5 months (Quad.: 120.6 ± 24.5% in the C-BTB vs. 70.0 ± 12.3% in the I-BTB; *p* < 0.001, Ham.: 95.6 ± 17.2% in the C-BTB vs. 97.4 ± 23.9% in the I-BTB; *p* = 0.055) and 12 months (Quad.: 96.9 ± 12.4% in the C-BTB vs. 76.0 ± 17.6% in the I-BTB; *p* = 0.018, Ham.: 95.3 ± 13.3% in the C-BTB vs. 97.9 ± 12.8% in the I-BTB; *p* = 0.74) postoperatively

**Fig. 2:**
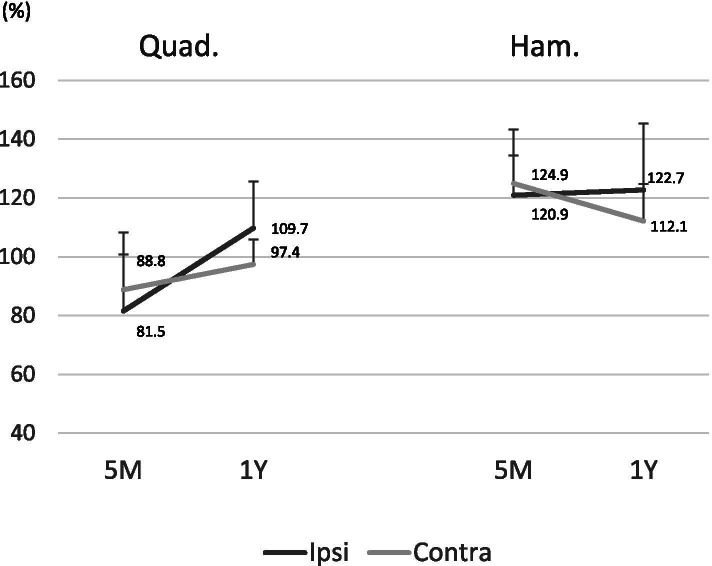
Muscle strength recovery of the BTB reconstructed knees in relation to the preoperative normal knees was investigated. No significant difference was noted in the quadriceps muscle strength between the two groups at 5 months (Quad.: 88.8 ± 19.5% in the C-BTB vs. 81.5 ± 19.1% in the I-BTB; *p* = 0.38, Ham.: 124.9 ± 9.5% in the C-BTB vs. 120.9 ± 24.0% in the I-BTB; *p* = 0.48) and 12 months (Quad.: 97.4 ± 8.4% in the C-BTB vs. 109.7 ± 18.3% in the I-BTB; *p* = 0.12, Ham.: 112.1 ± 12.8% in the C-BTB vs. 122.7 ± 34.6% in the I-BTB; *p* = 0.12) after surgery

**Fig. 3:**
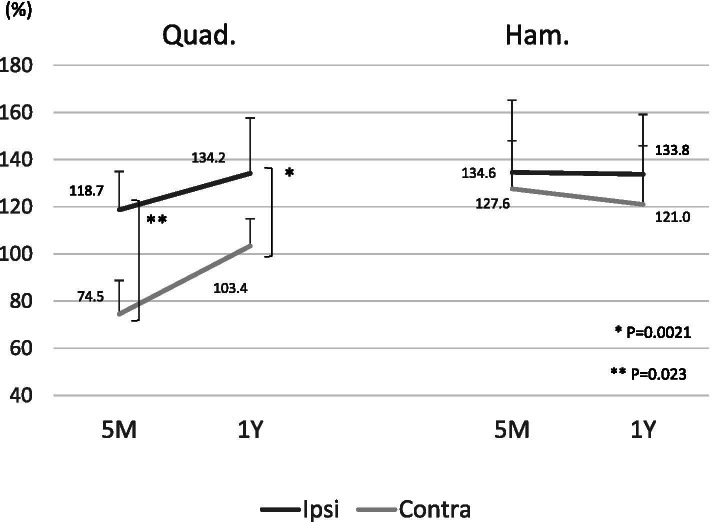
Muscle strength recovery of the non-reconstructed knees in relation to the preoperative normal knees was investigated. Quadriceps muscle strength was significantly weaker in the C-BTB group at 5 months after surgery (Quad.: 74.5 ± 14.4% in the C-BTB vs. 118.7 ± 27.4% in the I-BTB; *p* = 0.0021, Ham.: 127.6 ± 20.4% in the C-BTB vs. 134.6 ± 26.3% in the I-BTB; *p* = 0.60). Moreover, the quadriceps muscle weakness continued at 12 months (Quad.: 103.4 ± 11.5% in the C-BTB vs. 134.2 ± 22.9% in the I-BTB; *p* = 0.023, Ham.: 121.0 ± 24.9% in the C-BTB vs. 133.8 ± 25.7% in the I-BTB; *p* = 0.40)

The return-to-play time was significantly earlier in the C-BTB group than in the I-BTB group (6.5 months in the C-BTB group vs. 8.0 months in the I-BTB group; *p* = 0.021). Clinical outcomes at 1 year after surgery were not significant different according to the Tegner activity scales and the tibial anterior translation measured using the KT-2000 arthrometer. Concerning the occurrence rates of second ACL injuries, graft reinjury was 6.7% (1/15) in both groups, and the contralateral ACL injury was 6.7% (1/15) in the I-BTB group, with none of the patients in the C-BTB group deploying this injury within the follow-up period (Table 2). Concerning the comparing muscle strength between the reconstructed knee and un-reconstructed knee, the quadriceps muscle strength in the C-BTB group is significantly higher than that in the I-BTB group at 5 months and 12 months postoperatively. In contrast, hamstring muscle strength was not significantly different between the groups (Fig. 1). The postoperative chronological quadriceps and hamstring muscle strength changes in the reconstructed knees compared to the preoperative normal limb were not significantly different between the two groups (Fig. 2). However, muscle strength in non-reconstructed knees (graft donor knee in the C-BTB group and uninvolved intact knee in the I-BTB group) compared to the preoperative normal limb was significantly weaker in the C-BTB group than in the I-BTB group at 5 months (*p* = 0.0021) and 12 months (*p* = 0.023) after surgery in the quadriceps. In contrast, hamstring muscle strength was not significantly different at either 5 months (*p* = 0.33) or 12 months (*p* = 0.74) after surgery (Fig. 3). Twenty percentage (3/15) of the patients in the C-BTB group required local anesthetic injection therapy before or after return to playing sports due to the graft harvested site pain, yet none of the patients in the I-BTB group required anesthetic injections (*p* = 0.069).

**Table 2:** Clinical data after surgery

Group		Contralateral BTB	Ipsilateral BTB	*p* value
		(n=15)	(n=15)	
Time of return-to-sports	(median [ min.-max.])	6.5 [5-8.5]	8 [6-11]	0.012
Tegner activity scale	(median [ min.-max.])	9 [6-10]	9 [6-10]	1
Lachman				
negative	(n (%))	13 (86.7)	13 (86.7)	1
glide		2 (13.3)	2 (13.3)	
gross		0 (0)	0 (0)	
Pivot shift				
negative	(n (%))	12 (80.0)	12 (80.0)	1
glide		2 (13.3)	2 (13.3)	
gross		1 (6.7)	1 (6.7)	
Anteror translation difference (mm)	(mean ± SD)	1.3±1.2	1.9 ± 1.9	0.36
Second ACL injuries				
graft re-rupture	(n (%))	1 (6.7)	1 (6.7)	
contralateral ACL injuries	(n (%))	0 (0)	1 (6.7)	

*BTB* Bone-tendon-bone

## Discussion

The most important aspect of this study was to investigate postoperative muscle strength whether the contralateral BTB reconstruction meets the early return-to-sports criteria. To date, no previous reports have referred to muscle strength at the early stages within 1 year after surgery of C-BTB reconstruction. In this study, the patients who underwent C-BTB ACL reconstruction shown that the 120.6% of quadriceps and 95.6% of hamstring muscles strength of the reconstructed knee compared with the non-reconstructed knee at 5 months after surgery. From this rapid muscle strength recovery, the C-BTB approach may superficially seems to be an acceptable modification to ACL reconstruction to allow patients to return to play sports in the early stages. However, we note that the quadriceps muscle strength of the non-injured knee decreased by 74.5% in the graft harvested knee and 88.8% in the ACL-reconstructed knee at the same time. Early muscle strength recovery of the reconstructed knee compared to the none-reconstructed knee is due to bilateral quadriceps muscle strength deficits. We doubt that this technical modification secures a safe early return to sports with bilateral quadriceps muscle weakness. From the results, we have not attempted primary ACL reconstruction using a BTB autograft from the contralateral knee since 2015.

In general, the concept of rehabilitation in patients who underwent ACL reconstruction has antagonistic effects. One is the temporal postoperative rest of the reconstructed knee to promote graft and bone tunnel healing. The other is early knee ROM exercises and muscle training to build up to the preoperative condition and prevent graft site morbidities. Theoretically, the C-BTB ACL reconstruction has an advantage over the conventional I-BTB ACL reconstruction by facilitating postoperative rehabilitation by dividing the duplicative antagonistic effect on a single knee into two separate knees. Shelbourne et al. developed the advantages of the C-BTB approach of ACL reconstruction to allow patients to return to playing sports earlier by restoring ROM and muscle strength [14, 16]. Benner et al. also reported the advantages of the C-BTB approach in terms of postoperative complications [3]. They concluded that the incidence of infection or patellar tendon rupture was not different between the ipsilateral and contralateral BTB reconstruction groups. However, patients with complications after ACL reconstruction in the contralateral group may have less difficulty in obtaining full ROM than those in the ipsilateral reconstruction group.

In contrast, Mastrokalos et al. [10] reported that contralateral grafts appeared to offer no significant advantages over ipsilateral grafts. There was no tibial translation difference measured using the KT-2000 arthrometer and no difference in the Cincinnati Knee Rating System and Tegner activity scores. In thier contralateral BTB group, 70.9% of the patients experienced kneeling pain in the donor knee, whereas in their ipsilateral BTB group, 69.2% of the patients experienced kneeling pain in the donor knee, showing no significant difference between the two groups. In addition, contralateral ACL reconstruction did not result in a shorter return-to-play time compared to ipsilateral ACL reconstruction. Therefore, they concluded that the symptoms related to morbidity of the donor site pain was transferred to the uninvolved knee without any advantages.

With regard to the graft site pain in this study, 20% of the patients in the C-BTB group required anesthetic injections at the donor site to control anterior knee pain when they returned to playing sports; in contrast, none of the patients in the I-BTB group required anesthetic injection therapy. Mastrokalos et al. [10] evaluated the postoperative donor site morbidity. Kneeling pain in the donor knee was experienced by 69.2% and 70.9% of the patients in the ipsilateral and contralateral BTB groups, respectively. Although the overall comparison of the donor site pain rates between the two groups was not statistically significant, in the subgroup analysis, the rate of severe kneeling pain in the donor knee in the patients in the contralateral BTB group was significantly higher (27.1%) than that in the ipsilateral BTB group (9.6%) (*p* = 0.02). Rubinstein et al. [14], referring to the donor site morbidity using contralateral BTB grafts in mostly revision ACL reconstruction cases, reported that the most common complication was intensive donor site patellar tendon pain. They added that the transient pain was unresolved even at 1 year after surgery, while postoperative donor site pain decreased sequentially.

In contrast, Shelbourne et al. reported that no difference in kneeling and severity of pain in the International Knee Documentation Committee Subjective Knee Form subscores between the contralateral and ipsilateral BTB groups (*p* = 0.686 in kneeling and *p* = 0.564 in severity of pain) [13, 15]. They concluded that failure to prevent anterior knee pain was mostly due to extension deficit of the knee during the preoperative and postoperative periods [13, 15]. Although we also focused on the ROM exercise and muscle training from pre- to postoperative rehabilitation to minimize extension deficit, thus preventing anterior knee pain according to this recommendation, 20% of the contralateral BTB reconstruction still required local anesthetic injection therapy to reduce severe knee pain. We believe that the knee pain linked to the quadriceps muscle weakness is a significant reason for the donor site knee in the contralateral BTB reconstruction as the non-ACL injured limb should be the dominant leg in the early stage of return to sports. For these reasons, we have abandoned the adaptation of the contralateral BTB graft modification to achieve early return to sports since 2015.

As another approach to reduce donor site pain, the technique of refilling with a substitute material into the bony defect after harvesting the BTB graft was introduced [8, 11]. Refilling the void with a substitute material reduced graft site kneeling pain and postoperative patellar crepitus compared to non-refilling techniques. A well-designed comprehensive rehabilitation program and graft harvesting technique will be useful for reducing pain.

As with all studies, the limitations of this study exist. First, this was a retrospective study with a small number of patients. Fifteen patients apparently lacked power for the analysis. However, because no patients have been performed using this modification technique presently, the study has stopped as a pilot study that had implied the limitation of this technique. Moreover, the small number of patients did not increase the risk of second ACL injuries that should be critically evaluated. Second, with regard to the donor site morbidity as a result of bone-patellar tendon-bone autograft harvesting, graft site pain is evaluated only based on the frequency of the injection treatments at the site. We did not compare the C-BTB group with the I-BTB group using a subjective or objective knee score. However, to the best of our knowledge, no other studies have evaluated quadriceps and hamstring muscle strength in the early period when the patients anticipated going back to their previous sports. This information will guide surgeons and patients when they prefer accelerated rehabilitation after ACL reconstruction via contralateral BTB autografts.

## Conclusions

ACL reconstruction via the C-BTB autograft indicated better quadriceps muscle strength from early stage after surgery compared with I-BTB ACL reconstruction. However, we should consider that muscle strength symmetry can result from simultaneous bilateral knee muscle weakness compared to the preoperative condition. The contralateral ACL reconstruction has limitations in resolving the early return-to-play issue securely.
